# Mindfulness on Symptom Control and Quality of Life in Patients in Palliative Care: A Systematic Review

**DOI:** 10.1177/10499091231190879

**Published:** 2023-07-19

**Authors:** Anastasiya Stadnyk, Hugo Jorge Casimiro, Paulo Reis-Pina

**Affiliations:** 1 Faculty of Medicine, 37811 University of Lisbon, Lisbon, Portugal; 2 Hospital Palliative Care Team, Setúbal Hospital Centre, Setúbal, Portugal; 3 Bento Menni’s Palliative Care Unit, Casa de Saúde da Idanha, Sintra, Portugal

**Keywords:** mindfulness, palliative care, quality of life, symptom assessment, systematic review, terminal care

## Abstract

**Introduction:**

Palliative care is a medical and humanitarian approach that improves the quality of life of patients, and their families, who are facing problems associated with chronic and life-threatening illnesses. Few studies have evaluated the effectiveness of mindfulness-based interventions for terminally ill or incurable patients. The aim of this study was to systematically review the literature on the effect of mindfulness-based interventions on symptom control and quality of life in patients in palliative care.

**Methods:**

PubMed, Web of Science and Cochrane databases were searched for articles, published between January 2017 and December 2022, in English, including randomized controlled and clinical trials. *Participants*: terminally ill or incurable patients. *Interventions*: any mindfulness-based intervention. *Comparators*: any. *Outcomes*: symptom control and quality of life. The risk of bias was analysed through Cochrane’s ROB-2 tool.

**Results:**

Eight studies were included involving 609 patients and 75 dyads patients-spousal caregivers. The overall risk of bias was low to moderate. Mindfulness-based interventions are helpful in managing suffering, anxiety and depressive symptoms, fatigue, insomnia, drowsiness, appetite, and spiritual well-being.

**Conclusion:**

Mindfulness-based interventions control several symptoms and improve spiritual quality of life in patients in palliative care. Additionally, their informal caregivers also benefit from these interventions. Future trials are crucial to investigate other effects of mindfulness-based interventions, and their long-term benefits, in patients in palliative care.

## Introduction

### Rationale

The literature on palliative care (PALC) shows a vast boost in the emergence of non-pharmacological interventions to specifically address psychosocial and spiritual needs of terminally ill patients. Aside from other already known psychosocial PALC interventions such as dignity therapy, life review techniques and meaning-based interventions, mindfulness has too been proposed as a promising mechanism in the psychological coping with advanced or terminal illnesses.^1^

Despite deriving originally from Buddhist traditions, its low risk and ease of use made mindfulness widely accepted in various areas of modern health care as a secular training program.^2^ However, despite of its promising potential, only few studies have yet evaluated the effectiveness of mindfulness-based interventions (MBI) for patients with severe illnesses requiring specialised PALC.

When testing MBI, different investigators and health professionals came to divergent conclusions. Even though there are multiple studies that show convincing beneficial effects of mindfulness on quality of life (QOL), anxiety, depression, insomnia, stress, fatigue, physiological and cognitive functioning in the PALC population, other studies identify a few limitations to the approach and opposite results.^3^ Considering the both fragile and rapidly changing physical states of end-stage disease patients, PALC interventions should be brief and flexible. This might explain the findings of problem of adherence to treatment manuals together with the small magnitude and short endurance of effects on QOL.^3^

Succinctly, there has not been established an ultimate and assured conclusion about the feasibility and efficacy of mindfulness in the PALC setting. To analyse the presently available data, further investigations and review of evidence are imperative.

### Objectives

The aim of this study was to systematically review the literature on the role of mindfulness in PALC. Since mindfulness is a recent model of psychosocial care in the palliative medicine setting, this research reviewed the literature on the contribution of mindfulness in 1) providing symptom control; and 2) improving QOL in patients in PALC.

## Methods

### Eligibility Criteria

We selected articles that included: ***Participants-*** terminally ill or incurable patients. ***Interventions-*** any MBI. ***Comparators-*** any. ***Outcomes-*** symptom control and QOL.

### Information Sources

The PubMed, Web of Science and Cochrane databases were searched for articles published between January 1, 2017, and December 31, 2022. Each source was last searched or consulted on January 13, 2023.

### Search Strategy

The search included free-text terms and was done using these specific database headings: (“Palliative care” OR “terminal care” OR “end of life”) AND (mindfulness OR mindful*). Filters for randomized controlled trials (RCT) and clinical trials were applied.

### Selection Process

In the first analysis, evidence was chosen by reading and analyzing the titles and abstracts. The first author was responsible for that. Afterwards, a list of potentially useful articles was compiled, followed by a full-text analysis performed by 2 independent reviewers. Disagreements regarding study selection and data extraction were resolved by discussion and consensus between authors. No automation tools were used in the process.

### Data Collection Process

Two independent reviewers collected data from each report. The original authors were not contacted to obtain or confirm the data. No automation tools were used in the process.

### Data Items

Data were sought for 2 outcomes, symptom control and QOL, which could be studied alone, combined, or associated with other health-related outcomes. All results that were compatible with each outcome domain in each study were sought (for all measures).

Data were also sought for other variables, such as: a) article’s characteristics (e.g., authors, country of origin, year of publication); b) study design; c) objectives; d) population; e) intervention; f) comparator or control; and g) main outcomes. All these variables were included in a data-charting form that is shown in Table 1.

**Table 1.** Characteristics of the Individual Studies (n = 8).

Study; Country; Year	Study Design	Objectives	Population	Intervention	Comparator	Main Outcomes	Observations
Beng TS, et al^6^; Malaysia; 2019	Parallel-group, non-blinded, randomized controlled trial	To study the efficacy of a single session of MBI on the reduction in perceived suffering and Bispectral Index Score value	40 adult PALC in-patients with an overall suffering score ≥4 on suffering pictogram	−20 people: 12 females; 56.9 years (mean). −20-minute mindful-breathing session.	−20 people: 12 females; 58.1 years (mean). −20-minute supportive listening session.	-Suffering and pain scores: There were no differences between intervention and control (P > .05). -Bispectral index score value was different between intervention and control at T2, T3, T4 and T5 (all P < .001).	The Bispectral Index score is a validated monitoring device that measures the level of consciousness through electro-encephalographic signal.
Teo I, et al^12^; USA and Singapore; 2020	Pilot randomized controlled trial	To examine pre-treatment to post-treatment outcomes for distress, pain, and fatigue compared MBI sessions and control	85 women with stage IV breast cancer from outpatient oncology clinics from the USA (n = 40) and Singapore (n = 45)	-Four, 1-hour in-person individual sessions within 8 weeks. -USA patients (n = 15, mean age = 60 years). -Singapore patients (n = 19, mean age = 55 years).	USA (n = 19) and Singapore (n = 19)	-Intervention adherence criterion of 75%. -Medium effect sizes were found for anxiety, depression, and fatigue in the USA sample, and small effect sizes were found for anxiety, depression, and pain disability in the Singapore sample.	-Only 72 patients completed the study. -The MBI practice was “cognitive behavioural therapy with mindfulness and values-guided principles”. -The hospital anxiety and depression scale.
Warth M, et al^3^; Germany; 2020	Randomized crossover trial	To evaluate the stress-reducing effects of a brief, standardised MBI	42 adult PALC in-patient treatment; 36 patients completed both sessions; 69% were female; 65.88 years (mean)	20-minute short breathing exercise and guided body scan meditation for supine positions plus 40 minutes rest in a comfortable position in bed	Resting position for the entire 60 minutes	-For well-being and stress, MBI initially had a beneficial effect which attenuated towards the end. -The linear (P = .03) and quadratic effects (P = .02) for stress differed from the control, while the improvements in well-being did not.	
Milbury K, et al^10^; USA; 2020	Three‐arm pilot randomized controlled trial	To compare couple-based mindfulness vs supportive-expressive and usual care; arm targeting psycho-spiritual distress.	−75 adult dyads (patient and caregiver). -Patients with stage IV lung cancer (mean age = 65.0 years). -Spouses’ mean age = 63.9 years. −51% of patients and 51% of spouses were female.	-One 60-minute session once per week for 4 weeks. -MBI group focused on mindfulness exercises. -Supportive-expressive group focused on discussing cancer-related concerns.	Usual care provided by their health care team	-Patients and spouses rated the MBI beneficial (patients P = .003; spouses P = .005). -Depressive symptoms at 3-month follow-up: A) For patients- MBI vs usual care (P = .05) and MBI vs supportive-expressive (P = .07); B) For spouses- significant group main effect (P < .05), with difference between MBI and usual care (P < .01).	-The center of epidemiologic studies - depression scale was used. -Cancer-related stress symptoms and spiritual well-being: no significant group differences for patients or spouses.
Look ML, et al^7^; Malaysia; 2021	Parallel-group open-label randomized controlled trial	To determine the efficacy of a single session of 20-minute mindful-breathing in alleviating multiple symptoms.	40 adult PALC patients at least 1 moderate to severe symptom	-Four 5-minute breathing exercises done consecutively. -Symptom changes assessed.	-Social conversation. -Symptom changes assessed.	Improvement in drowsiness (P = .037), appetite (P = .017), depression (P = .004), anxiety (P = .02) and general well-being (P = .003) in the intervention group.	-The edmonton symptom assessment scale was used. -No statistical significance for the other symptoms.
Yik LL, et al^8^; Malaysia; 2021	Single-centre, parallel-group, randomized controlled trial	To investigate the effect of 5-minute mindfulness of peace on suffering and spiritual well-being.	60 adult PALC patients; 57 years (mean); with an overall suffering score of ≥4/10 on suffering pictogram.	−20 people (16 females). -Focus on the feeling of peace for 5 minutes, following a script with mindfulness practices.	−20 people (12 females). −5 minutes of active listening and answering about their life experiences.	In the MBI arm: reduction in the overall suffering (P = .001); rise in spiritual well-being (P = .004); reduction in worry (P = .014), non-acceptance (P = .025) and anger (P = .003) subscales; increase in meaning (P = .004) and faith (P = .026) subscales.	-The functional assessment of chronic illness therapy - spiritual well-being 12 scale was used.
Lim MA, et al^9^; Malaysia; 2021	Parallel-group, blinded, randomized controlled trial	To test the effect of 5-minute mindfulness of love on suffering and the spiritual quality of life.	60 adult patients in a PALC unit with an overall suffering score of ≥4/10; Mean age = 56 years	30 people (24 females); 5-minute mindfulness of love session guided by a medical doctor.	−30 people (18 females); 5-minute session (supportive listening) by a medical doctor.	-Improvements in the overall suffering (P < .001), the total suffering score (P = .007), and the total spiritual well-being (P < .001) in the intervention group.	-The functional assessment of chronic illness therapy - spiritual well-being 12 scale was used.
Bower JE, et al^11^; USA; 2021	Three-arm, phase III randomized controlled trial	To test the efficacy of 2 behavioural interventions for younger breast cancer survivors with elevated depressive symptoms.	247 women (46 years, mean age) with breast cancer who underwent treatment at least 6-month ago with mild depressive symptoms.	-Mindful-awareness practices and survivorship education interventions were conducted in groups, which met in person for 2-hour sessions conducted weekly for 6 weeks	Control condition	-Depressive symptoms- MBI vs control post-intervention (P = .001), 3-month (P < .001), 6-month follow-up (P = .013). -Fatigue- MBI vs control post-intervention (P < .001), 6-month follow-up (P = .002). -Insomnia- MBI vs control post-intervention (P = .006), 3-month follow-up (P < .001), 6-month follow-up (P = .002).	-The center of epidemiologic studies - depression scale was used. -The patient health Questionare-8 was used. -Survivorship education vs control post-intervention (P = .007), 3-month follow-up (P = .003).

Abbreviations: FACIT-Sp-12, Functional Assessment of Chronic Illness Therapy - Spiritual Well-Being 12; MBI, Mindfulness-Based Interventions; PALC, Palliative care; USA, the United States of America.

### Study Risk of Bias Assessment

Cochrane tools, both for RCT and non-RCT, were used to assess the risk of bias. For each domain, judgement was done by interpreting the algorithms suggested by the Rob2 Cochrane tool.^4^ Two independent reviewers assessed each study. No automation tools were used.

### Effect Measures

We accepted for each outcome the effect measure(s) declared by the authors of each study and used them both in the synthesis and in the presentation of the results.

### Synthesis Methods

A meta-analysis was not performed. A meta-regression would have been impossible to execute due to a) the small number of studies; and b) the insufficient homogeneity of the subjects involved, interventions, and outcomes to provide a meaningful summary.

The evidence was presented in a narrative format.

To visually display results of individual studies and syntheses we prepared a data-charting form which is shown in Table 1.

## Results

### Study Selection

**Figure 1.**
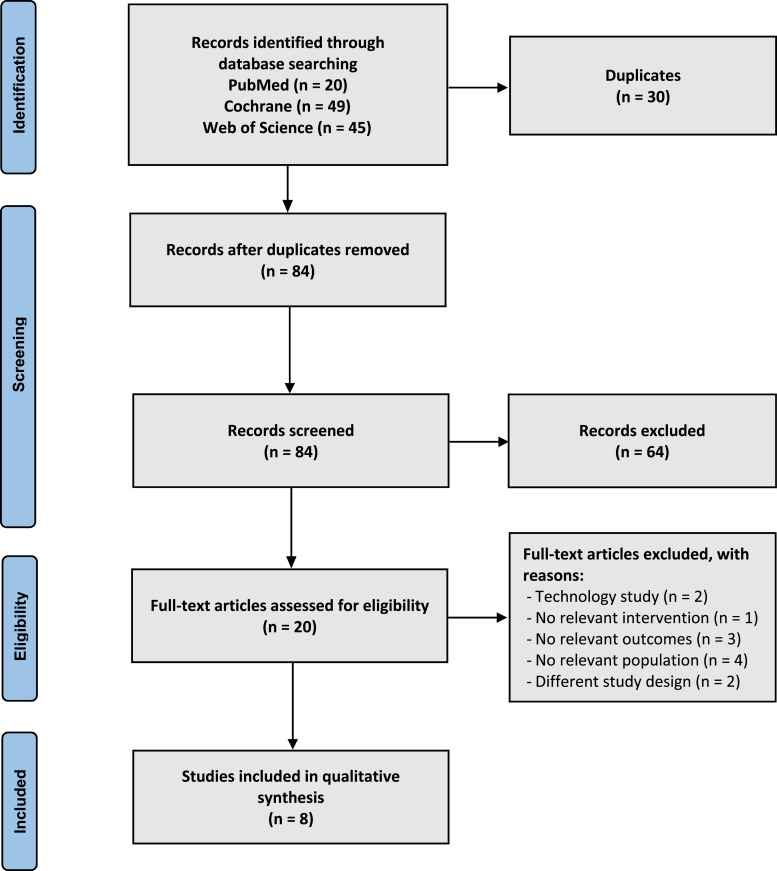
Flow diagram.

A total of 114 records were identified. No other records were added through manual search or other sources. After the removal of duplicates, 84 records remained. After the screening, 64 articles were excluded. Twenty full-text articles were assessed for eligibility; of these, 12 were excluded due to: no relevant population or intervention or outcomes (n = 8); different study designs (n = 2); and being technology-related studies (n = 2). Finally, 8 studies were included in this scoping review. A flow diagram of the study selection is presented in Figure 1, according to the “Preferred Reporting Items of Systematic Reviews and Meta-Analysis” statement.^5^

### Study Characteristics

Seven RCT and 1 randomized crossover trial were included. Studies were from Malaysia (n = 4),^6–9^ the USA (n = 2),^10,11^ Germany (n = 1),^3^ and Singapore/USA (n = 1).^12^ Participants were 609 PALC patients, together with 75 spousal caregivers. The characteristics of the studies are assembled in Table 1.

Beng TS, et al^6^, from Malaysia, in a RCT (parallel-group, non-blinded) studied the efficacy of “Mindful-Breathing” session, compared to “Supportive Listening” session, on the reduction in perceived level of suffering and the Bispectral Index Score (measures the level of consciousness) value among 40 PALC patients.^6^ They defended that when suffering reduces there is an increase in low-frequency electroencephalographic activities. The reduction of a patient’s arousal resembles mild to moderate sedation during the administration of anesthetic drugs.^6^

Teo I, et al^12^, from the USA and Singapore, in a pilot RCT, studied pre-treatment to post-treatment outcomes (distress, pain, and fatigue) comparing “Cognitive Behavioural Therapy with Mindfulness and Values-Guided Principles” sessions to control sessions, in 85 women with stage IV breast cancer.

Warth M, et al^3^, from Germany, in a randomized crossover trial, studied the stress-reducing effects of MBI (20 minutes short breathing exercise and meditation for supine positions, plus 40 minutes rest in a comfortable position in bed) compared to control (resting position for 60 minutes) in 36 patients in PALC.

Milbury K, et al^10^, from the USA, in a pilot RCT (three‐arm) targeting psychospiritual distress, compared “Couple-based mindfulness” to “Supportive-Expressive” and to usual care, in 75 adult dyads (patients with metastatic lung cancer and their spousal caregivers).

Look ML, et al^7^, from Malaysia, in a RCT (parallel-group, open-label) studied the efficacy of a single session of “Mindful-Breathing,” compared to “Social Conversation” in alleviating multiple symptoms in 40 patients in PALC.

Yik LL, et al^8^, from Malaysia, in a parallel-group RCT, studied the effect of “Mindfulness of Peace” on suffering and spiritual well-being, compared to “Active Listening and Answering” (about their life experiences), in 60 adult patients in PALC.

Lim MA, et al^9^, from Malaysia, in a RCT (parallel group, blinded), studied the effect of “Mindfulness of Love” on suffering and spiritual QOL, compared to “Supportive Listening” session, in 60 adults admitted to a PALC unit.

Bower JE, et al^11^, from the USA, in a phase III RCT (three-arm), assessed the efficacy of “Mindful-Awareness” practices, compared to “Survivorship Education” and to control, on depressive symptoms, in 247 women breast cancer survivors.

### Risk of Bias in Studies

**Figure 2.**
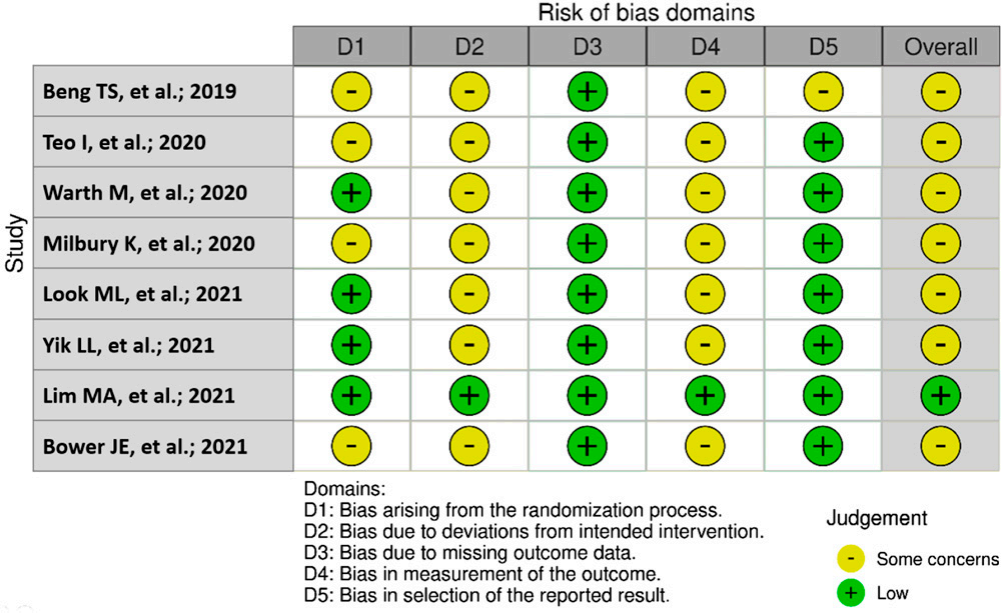
Risk of bias for randomized studies (n = 8).

The overall risk of bias was low to moderate (see Figure 2). The allocation sequence of the studies was considered of low risk, as the randomization was done by either using computer-generated random numbers,^6–9^ minimization randomization,^10^ or blocked randomization.^3,11^

Teo et al^12^ had 2 different randomization processes: blocked randomization for the intervention and sequenced opaque envelopes for the waitlist control condition.

More than half of the studies used an allocation concealment method;^3,7–9,12^ and the remaining did not provide any information.^6,10,11^

Lim et al^9^ was the only blinded trial. Bower et al^11^ stated that the unblinded nature of the assignment allocation constitutes a limitation in their trial. Look et al^7^ and Yik et al^8^ confirmed that blinding would have been impossible since the MBI required patient’s active participation.

There is no evidence in any of the studies that the methods of measuring or the measurement of outcomes differ between groups. However, Look et al^7^ admitted in their limitations that the unblinded trial context might have led to reporting bias as the outcome measure is subjective. We may extend these bias concerns to the rest of the non-blinded trials.

The risk of incomplete outcome data and selective reporting was low in all studies.

### Results of Individual Studies

In Beng et al's,^6^ the MBI group, vs control, showed suffering reduction (i.e., arousal reduction) during the first 5-minute session; at minute 5; during the subsequent 15-minute session; and at minute Twenty; all statistically significant (P < .001). There was no statistically significant difference between MBI and control for the perceived level of suffering or for pain scores.

In Teo et al’s,^12^ in the USA population, after MBI there was reduction in anxiety, depression, and fatigue scores, with medium effect sizes of .39, .43 and .50, respectively. In the Singaporean population, after MBI there was reduction in anxiety, depression, and pain disability scores, with small effect size of .23, .25 and .21, respectively.^12^

In Warth et al’s,^3^ the MBI group, vs control group, had a beneficial effect for subjective stress, with linear (P = .03) and quadratic effects (P = .02). The improvements in well-being were not statistically superior in the MBI group.

In Milbury et al’s,^10^ in the MBI group, vs Supportive-Expressive group and vs Usual Care, patients and spouses rated the overall intervention more beneficial (P = .003 and P = .005, respectively). For patients in the MBI group, Depressive symptoms improved after 3-month follow-up, vs Usual Care (P = .05) and vs Supportive-Expressive intervention (P = .07). For spouses, after 3-month follow-up, in the MBI group, Depressive symptoms improved, vs Usual Care (P < .01). There were no significant group differences for patients or spouses in cancer-related stress symptoms or in spiritual well-being.^10^

In Look et al’s,^7^ the MBI group, vs control, there was improvement in drowsiness (P = .037), appetite (P = .017), depression (P = .004), anxiety (P = .02) and general well-being (P = .003). No statistical significance was seen between groups for other symptoms such as pain, dyspnea, fatigue, or nausea/vomiting.^7^

In Yik et al’s,^8^ in the MBI arm, vs control arm, there was reduction in the overall suffering score (P = .001) as well as rise in spiritual well-being (P = .004). The improvement in spiritual QOL was due to greater increases in the meaning (P = .004) and faith (P = .026) and reductions in worry (P = .014), anger (P = .003) and non-acceptance (P = .025). There was no significant difference in the peace subscale between the MBI group and the control group.^8^

In Lim et al’s,^9^ in the MBI group, vs control group, there were improvements in the overall suffering score (P < .001), the total suffering score (P = .007), and the spiritual QOL (P < .001). The latter was due to greater reductions in worry (P = .027), anger (P = .047), non-acceptance (P = .018), and emptiness (P = .047).^9^

In Bower et al’s,^11^ prospectively, the MBI group, vs control, showed improvement in: 1) Depressive symptoms both post-intervention (P = .001), after 3-month (P < .001), and 6-month follow-up (P = .013); 2) Fatigue both post-intervention (P < .001), and after 6-month follow-up (P = .002); 3) Insomnia both post-intervention (P = .006), after 3-month (P < .001), and 6-month follow-up (P = .002).

The main results of the individual studies are summarized in Table 1.

## Discussion

This systematic review found 8 studies that strength the evidence on the effect of MBI on suffering, anxiety, and depressive symptoms, and on the spiritual QOL in PALC patients. Other symptoms were also reduced with MBI, such as drowsiness and appetite; insomnia; and fatigue.

The reduction of anxiety and depressive symptoms was the major key finding in this review. Four trials found significant improvements and reduction of anxiety and depressive symptoms.^7,10–12^ There is evidence of immediate effects and effects at 3-month,^10,11^ and 6-month follow up.^11^ Additionally, there is evidence not only for PALC patients,^7,10,12^ but also for their spousal caregivers.^10^ In a review, Shennan et al^13^ analysed 13 studies (RCT and non-RCT, pre- and post-test designs and qualitative studies), reporting 5 different MBI, and found significant improvements in anxiety, depression and stress across all interventions. Ando et al^14^ also found significantly decreased (P = .004) in depressive symptoms after MBI in Japanese patients with cancer. Foley et al^15^ also found that the MBI group showed significant improvement in depression, anxiety and distress, post-intervention, as compared with the control group; plus, the control group showed similar changes after MBI. In Look et al's,^7^ in the MBI group vs control, there was improvement not only in depression (P = .004) and anxiety (P = .02), but also in general well-being (P = .003). Kingston et al,^16^ found significant improvements in anxiety post-MBI, compared to the control group, in cancer patients, but no change in depression scores; although, at 3-month follow-up, the MBI group reported significant improvements in both depression and anxiety. In Milbury et al’s,^10^ depressive symptoms improved after 3-month follow-up, in the MBI group vs Usual Care, both for patients (P = .05) and spouses (P < .01). In Bower et al^11^’s, depressive symptoms in the MBI group vs control improved, not only post-intervention (P = .001), but also at 3-month (P < .001) and at 6-month follow-up (P = .013). Steinhauser et al,^17^ in the USA, did not find significant improvements, comparing MBI to control, in anxiety and depression after 7-week follow-up 1;^17,18^ but there was an improvement of social well-being (P = .05). In addition to analyzing the effects of MBI vs control on 1 symptom or another, although certain confounding factors are identified and isolated, there is room for complexity and heterogenicity that comes from the individual, cultural, clinical, etc. characteristics. The multicenter study from Teo et al^12^’s shows that: there was a greater reduction in anxiety and depression, in the MBI group vs control, in the USA population than in the Singaporean population.

Four trials assessed the effect of MBI on the reduction of perceived level of suffering and reduction in pain scores in PALC patients. Two trials found evidence of a significant reduction of perceived suffering,^8,9^ 1 trial showed reduction in pain scores,^12^ while 1 found no significant differences for subjective level of suffering or for pain scores,^6^ in the intervention group vs control. In Teo et al^12^’s, the Singaporean population showed a reduction in pain disability scores, but with small effect size of .21, comparing MBI to control. In Beng et al^6^’s, the MBI group showed “objective” suffering reduction, i.e. arousal reduction (P < .001), but there was no statistically significant difference between MBI and control for the subjective level of suffering nor for pain scores. In the literature, we found 2 studies with opposite results. Tan et al,^19^ in a RCT with advanced cancer patients, found statistically significant reduction in the overall suffering score in the MBI group compared with the control group (P = .008). On the contrary, Guan et al,^20^ compared MBI to “Normal Listening” (control group) in a PALC setting (n = 60) in Malaysia, and found that, although “Mindful-Breathing” was a quick and easy therapy to administer, the reduction of pain did not reach statistical difference between groups. We may speculate that these results differ because measuring pain and suffering is a major challenge, since suffering is such a unique and personal experience.

In the literature, there is also evidence about the effects of MBI on suffering in informal caregivers of PALC patients. Tan et al,^21^ from Malaysia, in a study with 40 adult informal caregivers of patients in PALC showed that the reduction in suffering scores in the “Mindful-Breathing” group was significantly greater than the control group (“Supportive Listening”) after 20 minutes (P = .036). They also found that the reduction of arousal in the MBI group was significantly greater than in the control group after 20 minutes (P < .0001).

MBI, vs control, showed improvement in drowsiness (P = .037) and appetite (P = .017),^7^ and insomnia.^11^ The latter improves immediately after MBI vs control (P = .006), and its effects lasts for a 6-month period (P = .002).^11^ In this review we also found controversial results for fatigue control. No statistical significance was seen between MBI and control groups for fatigue in the Malaysian study of Look et al^7^ Nevertheless, Bower et al^11^ showed that MBI, vs control, reduced fatigue scores (medium effect sizes of .50), and the effect of MBI lasted even after 6-month follow-up (P = .002).

In this review we found evidence that, after MBI, there was an increasing of patients’ spiritual QOL. This spiritual well-being is due to the reduction of feelings such as worry, anger and non-acceptance,^8,9^ and to increased meaning and faith.^8^ In Milbury et al's^10^ there were no significant differences between the groups (MBI vs Supportive-Expressive/Usual Care) for the spiritual well-being of patients or spouses. Steinhauser et al^18^ did not find significant improvements with MBI in the spiritual well-being of patients after a 7-week follow-up. The effects of MBI on the spiritual well-being has been shown in other vulnerable populations such as cancer survivors,^22^ and people with chronic diseases like diabetes mellitus.^23^

Mindfulness alone is still scarcely researched, let alone its use in clinics and especially PALC. To keep its integrity and ensure their reliability, future studies should ideally follow consolidated standards of reporting trials recommendations, and apply adequate methods for generation of randomisation sequence, allocation concealment and blinding of participants, personnel, and outcome assessment. Mindfulness techniques should be standardised and clinically relevant outcomes for patients should be elected. Evaluation of the effects of interventions should be obtained in longer periods of follow up.^13^

This review has some limitations. One of them is the scarce sample size. All included studies diverged in method, MBI practices, sample characteristics and in key findings, which made it difficult to summarize results and provide a confident conclusion on the exact benefits of MBI in the PALC setting. From the assessment of risk of bias, we concluded that allocation concealment, measurement and report of outcomes all showed to be troublesome due to the behavioural nature of the trials. This constitutes a big limitation to the reliability of the evidence. Also, even though objective scales were used to measure suffering, anxiety and depressive symptoms, these domains remain difficult to measure and assess objectively.

## Conclusion

In this systematic review, despite the magnitude of evidence being small, we found that mindfulness benefits patients in PALC by easing their perceived suffering level, reducing anxiety and depressive symptoms and by improving patients’ spiritual well-being, as well as of their marital caretakers.

MBI should be considered and further investigated as an alliance and potential pillar in the palliative medicine. Nevertheless, high quality RCT are still necessary to rebuttal the real benefits of mindfulness in PALC.
